# First-Principles Exploration into the Physical and Chemical Properties of Certain Newly Identified SnO_2_ Polymorphs

**DOI:** 10.1021/acsomega.1c07063

**Published:** 2022-03-16

**Authors:** Kanimozhi Balakrishnan, Vasu Veerapandy, Helmer Fjellvåg, Ponniah Vajeeston

**Affiliations:** † Department of Computational Physics, School of Physics, Madurai Kamaraj University, Palkalai Nagar, Madurai 625021, Tamil Nadu, India; ‡ Center for Materials Science and Nanotechnology, Department of Chemistry, University of Oslo, Oslo 0371, Norway

## Abstract

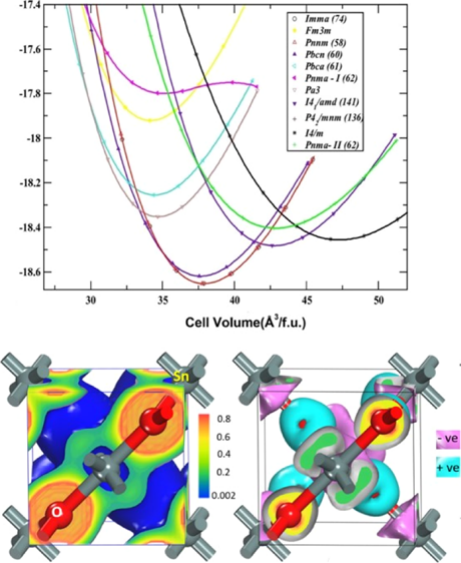

Tin dioxide (SnO_2_) is one of the transparent conductive oxides that has aroused the interest of researchers due to its wide range of applications. SnO_2_ exists in a variety of polymorphs with different atomic structures and Sn–O connectivity. However, there are no comprehensive studies on the physical and chemical properties of SnO_2_ polymorphs. For the first time, we investigated the structural stability and ground-state properties of 20 polymorphs in the sequence of experimental structures determined by density functional theory. We used a systematic analytical method to determine the viability of polymorphs for practical applications. Among the structurally stable polymorphs, *Fm*3̅*m*, *I*4_1_/*amd*, and *Pnma-II* are dynamically unstable. As far as we know, no previous research has investigated the electronic properties of SnO_2_ polymorphs from the hybrid functional of Heyd, Scuseria, and Erhzerhof (HSE06) except *P*4_2_/*mnm*, with calculated band gap values ranging from 2.15 to 3.35 eV. The dielectric properties of the polymorphs have been reported, suggesting that SnO_2_ polymorphs are also suitable for energy storage applications. The bonding nature of the global minimum rutile structure is analyzed from charge density, charge transfer, and electron localization function. The *Imma*-SnO_2_ polymorph is mechanically unstable, while the remaining polymorphs met all stability criteria. Further, we calculated Raman and IR spectra, elastic moduli, anisotropic factors, and the direction-dependent elastic moduli of stable polymorphs. Although there are many polymorphic forms of SnO_2_, rutile is a promising candidate for many applications; however, we investigated the feasibility of the remaining polymorphs for practical applications.

## 1 Introduction

Although oxides are insulators, some metal oxides like simple binary oxides, including ZnO_2_, In_2_O_3_, SnO_2_, and so forth, and complex oxides, like Zn–Sn–O and In–Sn–O, actually have properties of semiconductors as well as metals. SnO_2_ is a well-studied wide band gap semiconductor because of its excellent physical and chemical properties. SnO_2_ has received a lot of attention as a potential substitute for conventional titanium dioxide (TiO_2_) in dye-sensitized solar cells (DSSCs) because of its wide band gap, electron mobility, and excellent optical and chemical stability.^1^ Apart from this, SnO_2_ is a non-toxic, inexpensive, and high surface-to-volume ratio material.^2^ Currently, SnO_2_ is recognized for its properties as a most popular gas sensor^3−5^ and, most recently, in catalysis as a thin-film transistor,^6^ as well as a potential candidate for electrode materials for batteries.^7^ In the photovoltaic industry, SnO_2_ is a highly promising material to be used as a transparent conductive oxide. F-doped SnO_2_ has the highest work function compared to other transparent conductive oxide materials. It has the best contact with Si and forms a highly stable nanocrystal electron transport layer (ETL) in devices such as organic light-emitting diodes (OLEDs) and perovskite solar cells.^8,9^

The United States Geological Survey (USGS) reports that 31,000 tons of tin were produced in 2019, with China accounting for 27% of the total.^10^ Tin-based materials, especially SnO_2_, have attracted a lot of attention in the field of sensors because they have the most chemically and thermally stable oxidation states and are amazingly sensitive to various gas species. Even though SnO_2_ has been studied for a variety of applications, 37% of research has focused on its gas sensing property, with mixed oxides and TiO_2_ in the second and third places, respectively.^11^ Most of the research groups are concerned with the synthesis, characterization, and application of SnO_2_ as a gas sensor. According to a review of the literature, modified nanostructured SnO_2_ is used as an electrode for chemical sensors.^12^ Wang et al. demonstrated that when carbon derivatives are combined with SnO_2_ nanomaterials, their sensing performance improves. When compared to reduced graphene oxide (rGO), they demonstrated that the rGO/SnO_2_ composite is the greatest sensor of NO_2_.^13^ Therefore, SnO_2_ is an excellent material as a gas sensor for flammable and dangerous gases, such as hydrogen, NO_2_, acetone, CO, H_2_S, and methane.^14−16^

Many applications of inorganic crystals are derived from the crystal structure. SnO_2_ exists in a variety of polymorphic forms with varying utility. Experiments show that the SnO_2_ crystal lattice is highly pressure sensitive, implying that phase transition occurs.^17^ The phase transition of SnO_2_ follows the trend of phase transition of some other dioxides, such as SiO_2,_ MnO_2_, and RuO_2_. One of the most common SnO_2_ polymorphs is rutile.^18^ The majority of the experimental work has focused on the rutile phase because it is a naturally occurring form of SnO_2_.^19−21^ Recent developments in crystallographic techniques under high pressure have made it possible to investigate pressure-induced phase transitions. The pressure-dependent phase transition of SnO_2_ has been studied experimentally by J. Haines et al. However, a fundamental understanding of phase transition has been reported.^17^ The rutile polymorph of SnO_2_ underwent a phase transition to a CaCl_2_ polymorph at 11.8 GPa under hydrostatic conditions; the next transition was observed for the α-PbO_2_ polymorph beginning at above 12 GPa. It was also found that above 21 GPa, both the α-PbO_2_ and CaCl_2_ polymorphs transformed into a modified fluorite polymorph. Similarly, Erdem et al. theoretically investigated the transition pressures and phase sequences of SnO_2_ polymorphs.^22^ The authors discussed the transition pressures of the phases in the structural sequences of *P*4_2_/*mnm*–*Pnnm*–*Pbcn*–*Pa*3̅–*Pbca*–*Pnma* with pressures of 7.59, 11.50, 18.70, 25.69, 32.71, and 19.70 GPa, respectively. The authors also determined the mechanical properties of all polymorphs at different pressures, such as 0, 5, 10, 15, and 18 GPa. The authors concluded that as the pressure increases, the elastic stiffness constant for all polymorphs increases.^22^ Jiang et al. studied that the room-temperature phase transition of rutile-to-pyrite type occurs slowly around 18 GPa. This finding contradicts the findings of Ono et al. who found that, at 300 K, no rutile-pyrite type transformation occurred.^23,24^ This occurs due to the phase transition at room temperature, but it is rectified at high temperatures. There are several DFT studies available on SnO_2_, which are comprehensively reported by Das et al.^25^ In addition, there are many DFT works that have been carried out on SnO_2_.^26−30^

Further, Erdem et al.^31^ carried out a theoretical study of the electronic structure and elastic and thermal properties on seven known polymorphs of SnO_2_. However, in-depth studies on theoretical analysis of physical and chemical properties of SnO_2_ polymorphs are still lacking. In this paper, we suggested that 20 polymorphs of SnO_2_ and DFT calculations were used to perform an in-depth theoretical analysis of their properties for the first time. We verified the structural, dynamical, and mechanical stability of the involved polymorphs. The Raman and infrared spectra of SnO_2_ polymorphs were theoretically investigated for the first time. This paper presents an overview of the possible metastable phases of SnO_2_ and their stability in comparison to the reported stable experimental polymorphs and also analyzes their mechanical and dynamical stability.

## 2 Results and Discussion

### 2.1 Structural Properties

**Figure 1:**
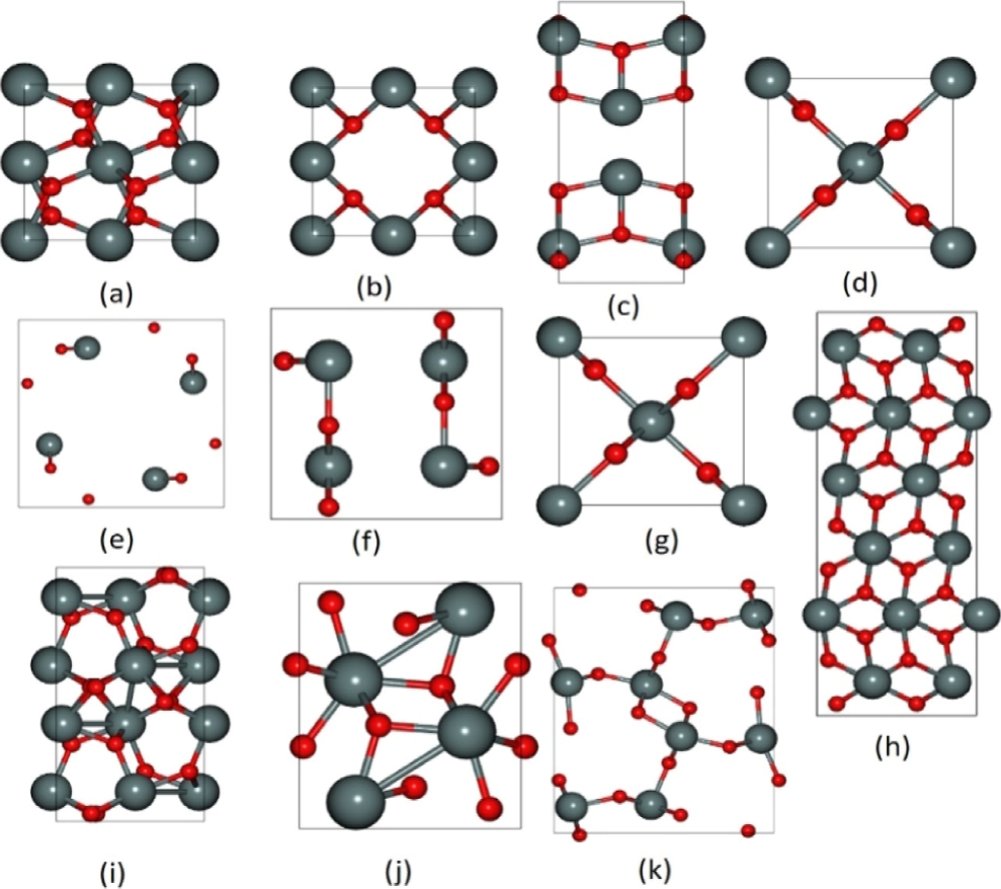
Optimized crystal structures of 11 polymorphs of SnO_2_, O atoms are in red color and Sn atoms are in gray color. (a) *Pa*3̅, (b) *Fm*3̅*m*, (c) *I*4_1_/*amd*, (d) *P*4_2_/*mnm* (e) *I*4/*m*, (f) *Imma*, (g) *Pnnm*, (h) *Pbcn*, (i) *Pbca*, (j) *Pnma-I* (k), and *Pnma-II* are the low-energy polymorphs that lie within 1 eV.

**Figure 2:**
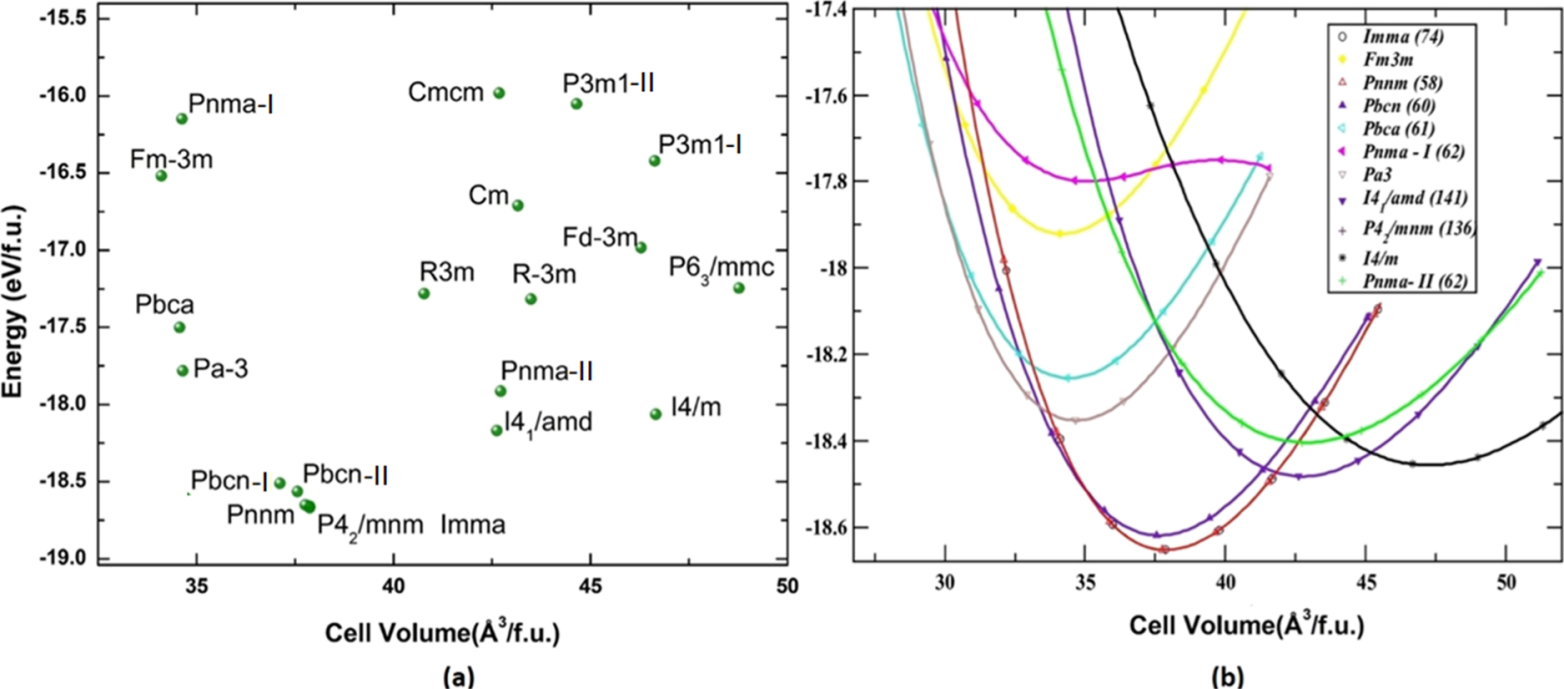
Calculated total energy as a function of the volume of different polymorphs of SnO_2_. (a) Total energy vs equilibrium volume of considered 20 polymorphs and (b) total energy as a function of volume for 11 low-energy polymorphs. All the energy volumes are normalized to one formula unit (f.u).

SnO_2_ exists in a number of polymorphic forms that have various utilities. Here in this study, we have investigated the relative stability of 20 polymorphs of SnO_2_, which are as follows: (the number of formula units, as well as the Materials Project ID, are given in parenthesis) *P*4_2_/*mnm* (2, 856), *Pa*3̅ (12, 697), *Pbcn-I* (12, 12978), *Fm*3̅*m* (4, 12979), *Pnnm* (2, 41011), *Pbcn-II* (12, 555487), *Pbca* (8, 560417), *Pnma-I* (4, 562610), *I*4_1_/*amd* (4, 755071), *Imma* (8, 1041584), *P*6_3_/*mmc* (6,1041584), *R*3*m-I* (12, 1044721), *Cmcm* (12, 1047381), *Fd-3m* (12, 1118394), *P*3*m*1*-I* (36, 5236), *Cm* (48, 5316), *Pnma*-II (24, 5659), *P*3*m*1-II (36, 9896), *R*3*m*-II (3, 11686), and *I*4/*m* (8, 15363). Only 11 low-energy SnO_2_ polymorphs were considered for further analysis, which are as follows: *Pa*3̅ (4, 697), *Fm*3̅*m* (4, 12979), *I*4_1_/*amd* (4, 755071), *P*4_2_/*mnm* (2, 856), *I*4/*m* (8, 15363), *Imma* (8, 1041584), *Pnnm* (2, 41011), *Pbcn* (12, 555487), *Pbca* (8, 560417), *Pnma-I* (4, 562610), and *Pnma-II* (8, 5659). The crystal structures of these 11 polymorphs are given in Figure 1. Other polymorphs were excluded from this study because their energy volume curves are presented above the range of 1 eV from the minimum energy polymorph, as shown in Figure 2.

**Figure 3:**
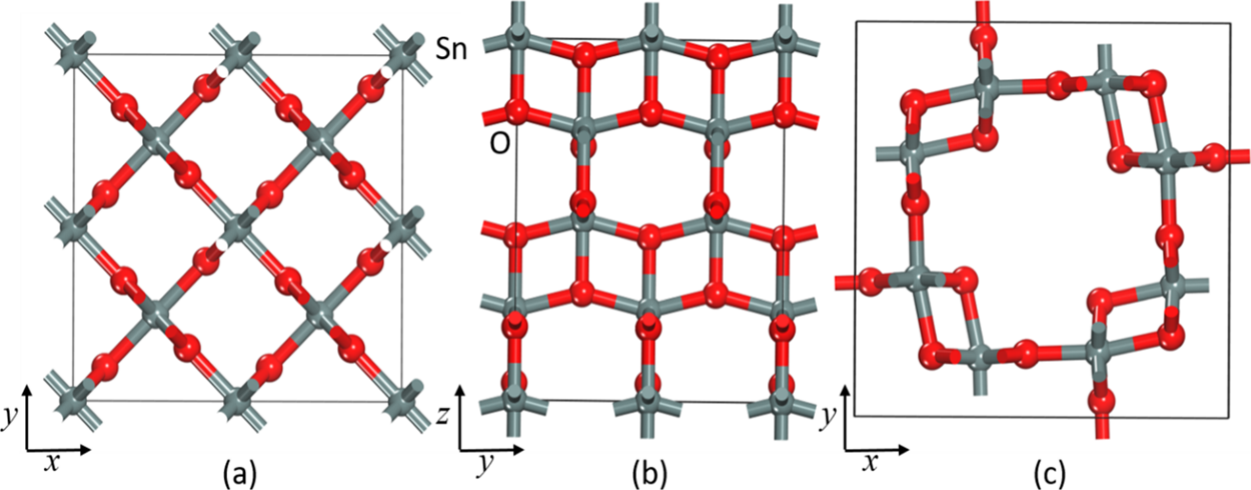
Optimized low-energy crystal structure of *P*4_2_/*mnm* (a) view along [001] and structures at the expanded lattice for *I*4_1_/*amd* (b) view along [100] and *I*4/*m* (c). To have a better view ability on the porous nature of the involved structures, the view angle is projected in different directions.

*Pa*3̅ and *Fm*3̅*m* polymorphs have a cubic structure, and *I*4_1_/amd, *P*4_2_/*mnm*, *I*4/*m*, and *Imma* are tetragonal, while the remaining polymorphs are orthorhombic. The structures of different polymorphs contain different SnO_2_ formula units, with the most studied experimental polymorph being rutile (*P*4_2_/*mnm*) containing two formula units, and *Pbcn* being the largest containing 12 formula units. The structural optimization was performed on the structures under zero pressure and zero kelvin, and the optimized structures were then used in subsequent calculations. 4 of the 11 phases were reported for the first time here, so we need to determine their relative stability with the global minimum polymorph by calculating total energy as a function of volume for 11 polymorphs. The lowest energy configuration is shared by three polymorphs: *Imma*, *P*4_2_/*mnm*, and *Pnnm*. It is important to highlight the fact that even though their E-V curves are similar to these three polymorphs, there is the smallest energy difference between them: 0.3 meV/f.u for *P*4_2_/*mnm* to *Pnnm* and 0.4 meV/f.u for *P*4_2_/*mnm* to *Imma*. As a result, by varying the temperature or pressure, one polymorph can be easily transformed into another. They also differ in *b*/*a* values due to their different lattice structures. The E–V curve for the *P*4_2_/*mnm* polymorph is the lowest among these three lowest energy shared polymorphs. With only minor distortions in the structures *Pnnm*, *P*4_2_/*mnm*, and *Imma* exhibit similar properties in our result. The energy minima of the three newly identified polymorphs, *I*4_1_/*amd*, *I*4/*m*, and *Pnma-II*, were observed in the expanded lattice when compared to *P*4_2_/*mnm*, indicating that we can experimentally stabilize these polymorphs. In general, the structures in the expanded lattice are loosely packed crystal structures that are porous in nature. In SnO_2_, *I*4_1_/*amd* and *I*4/*m* structures are much porous than the low-energy structure, as shown in Figure 3. Moreover, these three polymorphs may form experimentally in the nanophase. *I*4_1_/*amd*, *I*4/*m*, *Pbcn*, and *Pbca* are metastable polymorphs that were converted to low-energy polymorphs under certain experimental conditions such as pressure, temperature, and other experimental data. The equilibrium volume of the polymorphs varies from 32.8 to 46.8 Å^3^, whereas the energy of all 11 polymorphs are in an energy range of 0.98 eV. Also, nine polymorphs were above 100 meV higher than the low-energy configuration; hence, we omit 9 out of 20 polymorphs. In addition, our results are in accordance with the stated values of researchers that have previously found SnO_2_ polymorphs with rutile and CaCl_2_.^31^ The calculated lattice parameters and equilibrium volumes for various polymorphs are shown in Table 1. The calculated lattice constants of the rutile-SnO_2_ are *a* = *b* = 4.83 Å and *c* = 3.24 Å, and this seems to be in agreement with experimental and other theoretical reports.^17,24,30−32^ The calculated lattice constants of the polymorphs differ from 0.3 to 2% from the experimental and other theoretical findings.^25,32−38^ Especially, the lattice constants were compared with the high-pressure phase transition of the polymorphs.^32,35^ The third-order Birch–Murnaghan equation of state (BM-EOS) fits with the calculated energy as a function of volume data to obtain the bulk modulus of the polymorphs, as well as its first-order pressure derivatives, also shown in Table 1. The bulk modulus values were varying from 171 to 279 GPa and minimum in *Pnma-I* and maximum in *I*4/*m*; hence, the polymorphs of SnO_2_ are comparable with the modulus of cast iron. The bulk modulus calculated from B-M fit is consistent with the bulk modulus calculated from Voigt–Reuss–Hill approximation.

**Table 1:** Calculated Equilibrium Lattice Constants *a*, *b*, and *c* (Å), Equilibrium Volume *V*_0_ (Å^3^), Bulk Modulus *B*_0_ (GPa), and Its Pressure Derivative *B*_0_′, Band Gap (*E*_g_) eV, and Band Type of SnO_2_ Polymorphs

polymorphs	a (Å)	b (Å)	c (Å)	*V*0 (Å^3^)	*B*_0_ (GPa)	*B*_0_′	band type	*E*_g_ (eV)
*Pa*3̅ (205) pyrite	**4.99**			**139.05**	**229**	**3.1**	indirect	**3.35**
	4.90^17^				226^17^	3^17^		0.84^17^
	5.116^32^				216^32^	4.7^32^		3.45^35^
*Fm*3̅*m* (225) fluorite	**5.06**			**137.05**	**207**	**3.5**	direct	1.83
	4.99^35^				288^35^	6^35^		
	5.08^32^				204^32^	4.5^32^		
*I*4_1_/*amd* (141)	**3.98**	**3.98**	**10.19**	**172.53**	**121**	**2.8**	indirect	3.38
*P*4_2_/*mnm* (136) rutile	**4.83**	**4.83**	**3.24**	**76.30**	**190**	**3.3**	direct	**2.30**
	4.77^32^		3.212^32^		192^32^	4.8^32^		2.58^37^
	4.72^35^		3.19^35^		204^35^			2.72^37^
								3.49^38^
								0.83^32^
*I*4/*m* (87)	**10.75**	**10.75**	**3.22**	**396.68**	**279**	**2.7**	direct	**2.41**
*Imma* (74)	**6.48**	**6.83**	**6.83**	**302.64**	**201**	**3.3**	direct	**2.37**
*Pnnm* (58) CaCl_2_	**4.82**		**3.24**	**76.27**	**191**	**3.3**	direct	**2.34**
	4.827^17^		3.236^17^		173^17^	4^17^		0.89^22^
	4.808^32^		3.226^32^		195^32^	4.6^32^		3.66^35^
*Pbcn* (60) PbO_2_	**4.786**	**5.840**	**16.12**	**452.59**	**185**	**3.3**	direct	**2.31**
								1.16^17^
								3.76^35^
*Pbca* (61) ZrO_2_	**9.35**	**4.95**	**4.74**	**276.78**	**178**	**3.5**	indirect	**2.31**
	9.97^35^	5.11^35^	5.02^35^			4^35^		0.84^22^
Pnma-I (62) cotunnite	**5.21**	**3.20**	**6.21**	**155.72**	**249**	**4.8**	indirect	**2.15**
	5.33^35^	3.38^35^	6.67^35^		229^35^	4		0.52^35^
Pnma-II (62) cotunnite	**9.30**	**3.22**	**11.38**	**346.19**	**171**	**0.6**	indirect	**2.72**

### 2.2 Dynamical Properties

**Figure 4:**
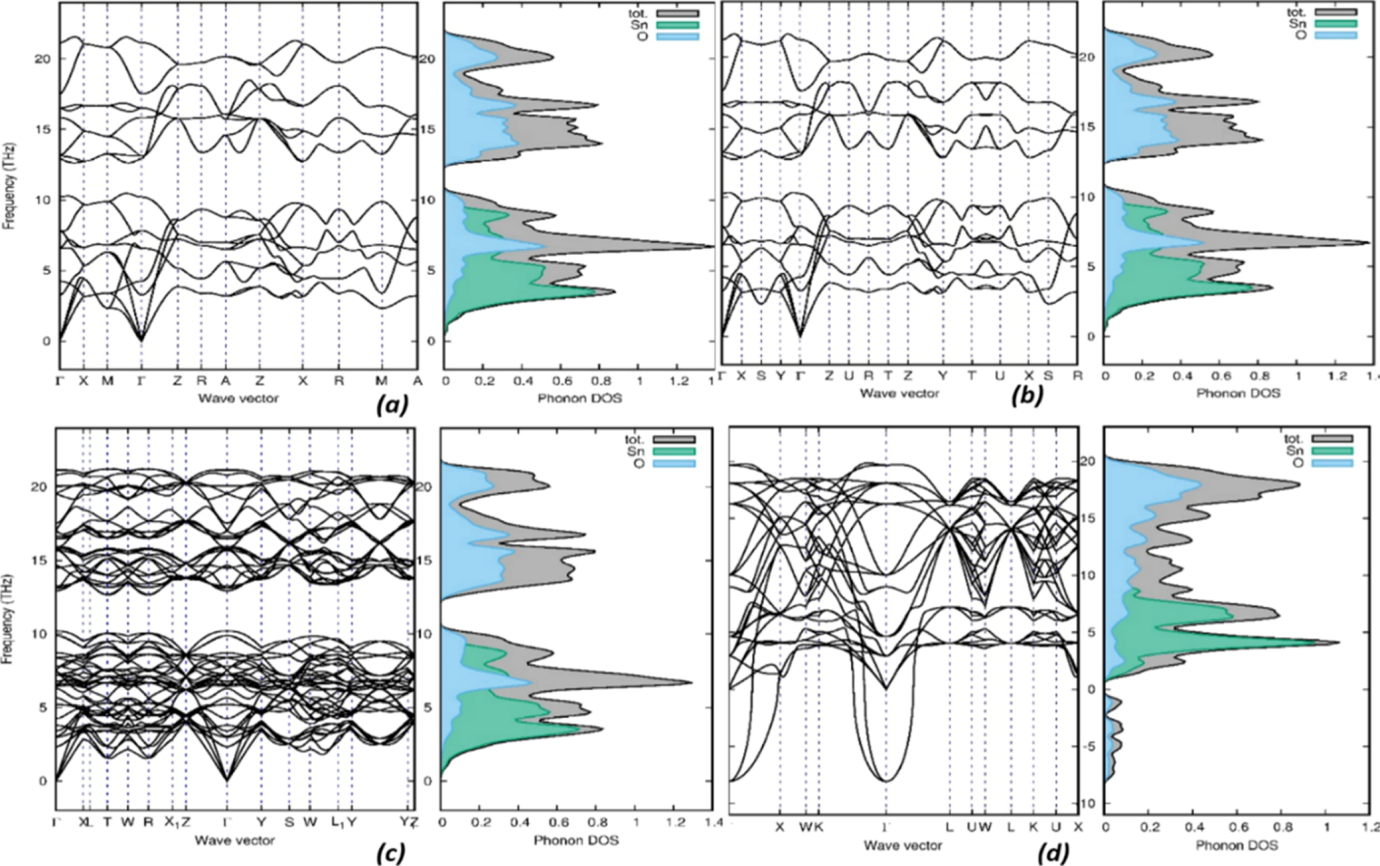
At high symmetric points, the calculated phonon dispersion with phonon density of states of the low-energy polymorphs (a) *P*4_2_/*mnm*, (b) *Pnnm*, and (c) *Imma* with a dynamically unstable polymorph of SnO_2_ and (d) *Fm3̅m*.

PHONOPY is a computational pre-/post-processing tool for the calculation of lattice dynamic and vibrational properties of solids from first-principles.^39^ SnO_2_ is a polar crystal that exhibits the splitting (LO/TO splitting) of infrared (IR) active modes into longitudinal and transverse modes at Γ due to dipole moments. Here, we have used the frozen phonon method to find the lattice dynamic properties of the polymorphs at the equilibrium volumes. Figure 4 depicts the phonon dispersion curve for minimum energy polymorphs, including an unstable *Fm*3̅*m*; the remaining polymorphs are shown in Figure S1 in the Supporting Information. The results show that the cubic fluorite (*Fm*3̅*m*) structure has imaginary phonon frequencies, indicating that it is a dynamically unstable phase; the imaginary frequencies were accrued at Γ. As well as *Pnma*-II and *I*4_1_/*amd* also have an imaginary frequency at X, which seems to be dynamically unstable and may not form under any experimental conditions. Our results of phonon frequencies of the rutile, CaCl_2_, PbO_2_, pyrite, ZrO_2_, and cotunnite structures are in good agreement with those calculated by Erdem et al. as well as other theoretical and experimental findings.^31,40−42^ The rest of the polymorphs are reported here for the first time. In the orthorhombic *Pbca*, and *Pnma*-I SnO_2_ structures, there is no separation between the acoustic and optical branches. The acoustic branches of the polymorphs *Pa*3̅, *P*4_2_/*mnm*, *I*4/*m*, *Imma*, *Pnnm*, and *Pbcn*, lie in the range 0–11 THz, while optical branches are from 13 to 26 THz. The separation of the branch acoustics and optics is well suited for photovoltaic purposes. From the phonon density of states, it is clear that the mode of vibration of the polymorphs varies due to the crystal structure. The vibration of acoustical branches is mainly due to the Sn atom with a small contribution of the oxygen atom, which is observed from the phonon density of states. The optical branches arise from the oxygen atom hybridized with the Sn atom. In all the low-energy configurations, the phonon dispersion curves are well separated; hence, all these phases might have less thermal recombination loss. Among the 11 low-energy polymorphs, only 8 polymorphs are both structurally and dynamically stable, so in the following sections, we considered only the stable polymorphs.

### 2.3 Electronic Properties

**Figure 5:**
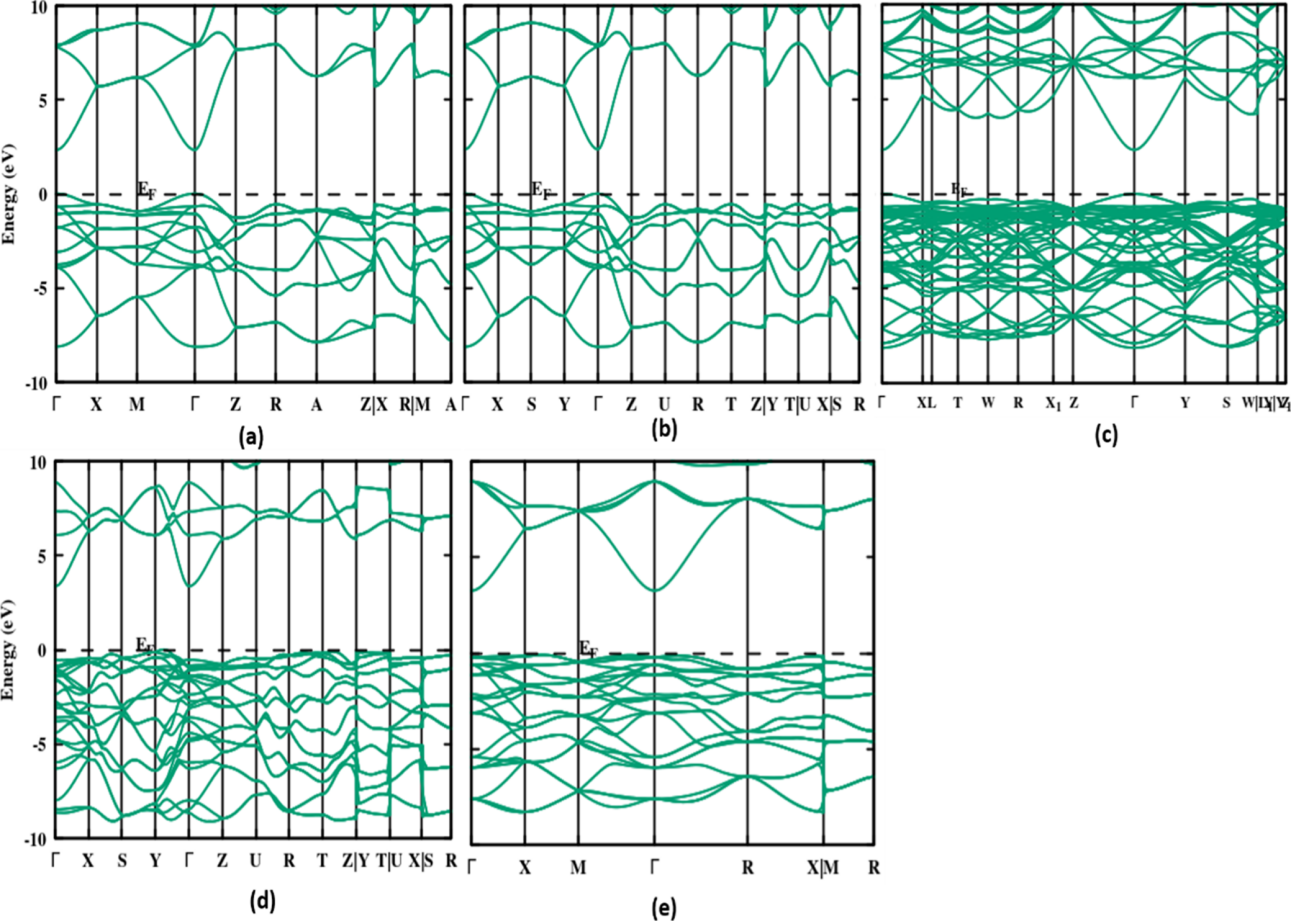
Calculated band structure at the HSE06 level for the low-energy polymorphs of SnO_2_, (a) *P*4_2_/*mnm*, (b) *Pnnm*, (c) *Imma*, as well as the low- and high-band gap polymorphs (d) *Pnma-I*, and (e) *Pa*3̅, the remaining phases are given in Figure S2 of Supporting Information.

In this section, we present the energy band structure and the electron density of states (DOS) of the polymorphs at equilibrium volume using the first-principles augmented plane wave (APW) method within the density functional theory. The band structures of the eight stable polymorphs are calculated for the highly symmetric points across the first Brillouin zone at zero pressure. Figure 5 depicts the band structures of the minimum energy polymorphs, as well as the highest and lowest band gap polymorphs, and the remaining polymorphs can be found in Figure S2. While analyzing the electronic properties, the SnO_2_ polymorphs show a semiconducting behavior, the corresponding band gap values are calculated by HSE06, and the type of band is given in Table 1. However, the polymorphs of SnO_2_ all fall into the category of wide band gap semiconductors, indicating that the material will be used in photovoltaics, photocatalysis, and a variety of other applications. When comparing the band structures of eight stable polymorphs, *Pa*3̅ (X−Γ), *Pbca* (Y−Γ), and *pnma-I*–SnO_2_ possess an indirect band gap, while the rest have a direct band gap. The direct band gap of the phases is observed at the high symmetric Γ-point.

**Figure 6:**
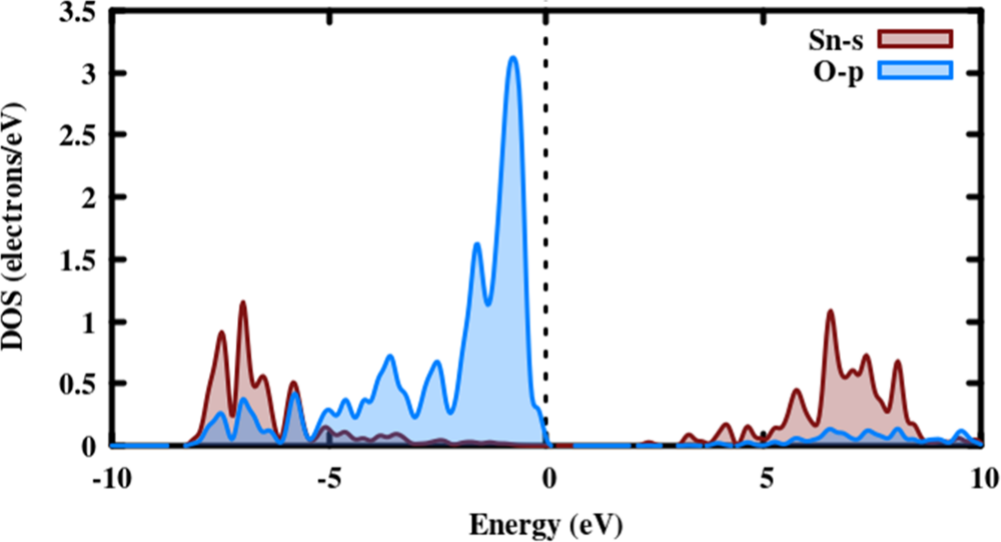
Partial DOS (PDOS) of rutile–SnO_2_ and O–P orbitals tends to produce the prominent peak in the valance band maximum, while the Sn-s orbital provides the maximum along the conduction band minimum.

The band gap of the polymorphs *Pnma-I* and *Pa*3̅ are at the two extremes, the band gap of *Pnma-I* is 2.15 and of *Pa*3̅ is 3.35 eV due to different crystal structures. Interestingly, by using the hybrid functional, the calculated band gap agrees well with the experimental results.^34,43^ Tingting et al. reported the band gap of rutile-structured SnO_2_ at different functionals, particularly at B3LYP and PBE0, which vary from 10 to 14% with our findings.^37^ Further, Gilani et al. investigated the band gap of rutile-SnO_2_ from the CASTEP code, which is 3.494 eV for the HSE06 functional.^38^ We also examine the band gap quantitatively, and it improved by 63% when we employed HSE instead of GGA with the reported values for the rutile polymorph.^22^ Other polymorphs are reported for the first time with a HSE06 functional. Thus, the usual problem of band gap discrepancy is overcome by using the hybrid functional. The band gap of the tetragonal and orthorhombic structures is almost similar; it is approximately 2.3 eV. The population of bands in all the cases, near the Fermi level (valence band maximum—VBM) between 0 and −8 eV is mainly due to O-2p-stated hybridizing with the Sn-5s states. The conduction band is dominated by 5s and 2p orbitals of Sn, with a small contribution from the O-2s orbital, as shown in Figure 6. Among the structurally stable polymorphs, some have a dense band population due to a higher population of free elections. For stable polymorphs, the bands were well separated especially at the gamma point, as observed for *Pa*3̅, *P*4_2_/*mnm*, *Pnnm* polymorphs.

### 2.4 Dielectric Properties

#### 2.4.1 Born Effective Charge Analysis

Polarization P induced on the atom by a lattice distortion is related to the Born effective charge (BEC) tensor Z*. In this work, the Z* of eight polymorphs involved is calculated and listed in Table S1 of Supporting Information. The interaction of lattice displacements and electrostatic fields is described by BEC. BEC predicts long-range Coulomb interactions for the splitting of LO and TO in phonons. Because of the structural symmetry, the charge tensor of each ion has anisotropic diagonal elements and finite off-diagonal elements. The formal valances of Sn and O ions are +4 and −2, respectively, and the charge tensors for pure ionic bonds are greater than the allowed values. Because the effective charge for a specific ion varies significantly, BEC is found to be quite sensitive to the ion position and crystal symmetry. We can see isotropy in the BEC of cubic structures (*Z*_xx_* = *Z*_yy_* = *Z*_zz_*) because all the diagonal elements have roughly the same value. However, for the tetragonal structures (*Z*_xx_* = *Z*_yy_*; *Z*_zz_*), two different values of BEC were *Z*_zz_* > *Z*_xx_* observed in the diagonal tensors, indicated by charge anisotropy. For orthorhombic structures, the degree of anisotropy increases, and all three diagonal tensors differ. The effective charge of Sn scatter ranged from 3.79 to 4.26, and the effective charge of the O atom ranged from −1.93 to −2.44, indicating that the charges changed as the Sn–O bond length changed. The effective charge differs from the principle value along the *c*-axis by only 6% for Sn atoms and 7% for O atoms. The computed BEC values differ from 0.2 to 3% with the reported values for the rutile SnO_2_ and other polymorphs of SnO_2_ reported for the first time.^31,42^

#### 2.4.2 Dielectric Constant

Dielectric materials play an important role in many electronic devices, including capacitors, computer memory (DRAM), sensors, and communication circuits. The calculated static dielectric constants are well matched with the experimental results for the naturally occurring SnO_2_ as 9.81 along the *a*-axis and 7.97 along the *c*-axis,^20,42,44−46^ as given in Table S1 in the Supporting Information. The dielectric constants of cubic structures are greater than the rest of the structures; therefore, the ionic polarizability is much greater for this phase. The material with a high dielectric constant can be used as a good capacitor; hence, the dielectric constant of SnO_2_ is also related to the dielectric constant of capacitors.^47^ As a result, SnO_2_ polymorphs could be used in storage applications. The anisotropy trend of the polymorph dielectric constants follows the anisotropy trend of the BEC.

### 2.5 Bonding Nature

**Figure 7:**
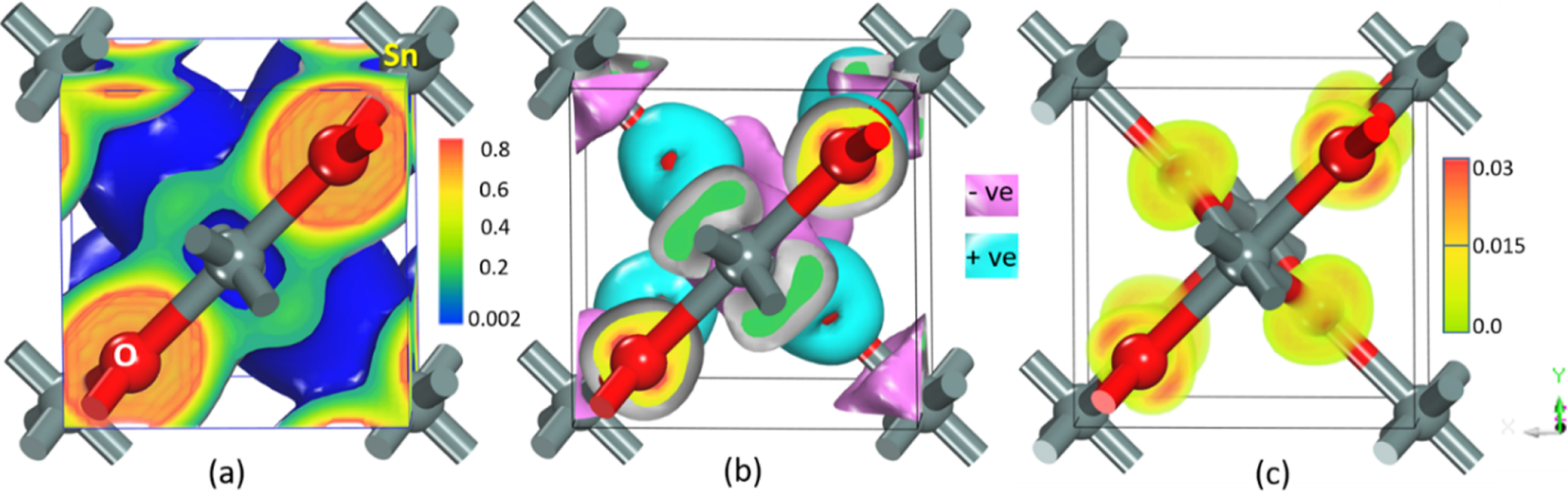
Calculated charge density (a), charge transfer (b), and electron localization function (c) plots for SnO_2_ in the rutile structure.

To gain a better understanding of bonding interactions, the calculated valence-charge-density distribution was used. All the polymorphs considered in this study are having almost a similar feature; hence, we have displayed only the charge density, charge transfer, and ELF plots of the lowest energy rutile polymorph is given in Figure 7, and it can be seen that the maximum charge density is accumulated at the O atom’s sites. There is no charge transfer between the cations indicated by blue. The fact that electrons are strongly localized on anions demonstrates the ionic nature of bonding in the rutile phase. All the stable polymorphs involved are ionic not just the rutile. In a practical system, the charge density is different because valance electrons take part in the calculations and leave out core electrons. When comparing this analysis with the experimental results, the d electron population of Sn ions also takes part in the bonding; hence, there are non-spherical valance electrons distributed around the cation.^48^ The bluish-green on the Sn site indicates that meager charges have been associated with Sn. Because the d-orbital is a core electron in the cation, the population becomes zero, and electrons from the 5s and 2p orbitals are transferred from Sn to the nearest O atom. We observed a quantitative charge transfer between the ions in Figure 7b, with 95% of electron transfer from Sn to O ion and only 5% from bonding between these, indicating that a dominant ionic character, which is similar to the charge density plot. The ELF confirms the charge localization only on anions, with electrons being depleted from the Sn site and accumulating on the O site. The accumulation of ions on the O site indicates the system’s ionic nature. A similar pattern has been observed in all the stable polymorphs of SnO_2_. The calculated bonding nature of rutile SnO_2_ in comparison to the reported values^49,50^ also is in good agreement with the PDOS analysis.

### 2.6 Mechanical Properties

#### 2.6.1 Mechanical Stability

So far, most theoretical studies are concerned with the mechanical and thermal properties of the rutile SnO_2_ alone. Only a very few reports are available on their mechanical properties. For example, Das et al.^51^ investigated the elastic properties of four polymorphs of SnO_2_ under different pressures and demonstrated the directional dependence of Young’s modulus of the four polymorphs. We determined the elastic constant by applying strain to a crystal and measured the associated stress in order to understand the mechanical stability of the identified stable polymorphs. In general, the elastic properties of a material are associated with the arrangement and strength of bonds between the atoms that make up the material. Hooke’s law related the elastic constants in a bulk solid with the linear response of stress tensor σ to external strain ε applied on a system. These elastic tensor components are calculated from the derivatives of energy as a function of the lattice strain as
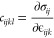
1

Table 2 gives the three independent elastic constants, *C*_11_, *C*_12_, and *C*_14_ calculated for the two cubic structures in their equilibrium lattice parameters. For the tetragonal system, there are six independent elastic constants *C*_11_, *C*_12_, *C*_13_, *C*_33_, *C*_44_, and *C*_66_, which should satisfy Born’s stability criteria.^52,53^ Whereas for the orthorhombic structures, there are nine elastic tensors *C*_11_, *C*_12_, *C*_13_, *C*_22_, *C*_23_, *C*_33_, *C*_44_, *C*_55_, and *C*_66_. Based on the Voigt–Reuss–Hill approximation,^54−56^ mechanical parameters, such as bulk modulus *B*, sheared modulus *G*, Young’s modulus *E*, and Poisson’s ratio ν, are determined. From the results of the elastic constants, Young’s modulus (*E*) and Poisson’s ratio (ν) of the polycrystalline materials are expressed as follows.
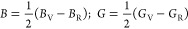
2
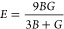
3
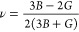
4

**Table 2:** Elastic Constants, Shear Modulus (*G*), Young’s Modulus (*E*), Bulk Modulus (*B*), and Poisson’s Ratio (σ) of SnO_2_ Polymorphs^a^

S. No	polymorphs	*C*_11_	*C*_12_	*C*_13_	*C*_22_	*C*_23_	*C*_33_	*C*_44_	*C*_55_	*C*_66_	σ	*A*^u^	*E* (GPa)	*G* (GPa)	*B* (GPa)
1	*Pa*3̅	**417**	**209**					**134**			**0.31**	**0.08**	**317**	**121**	**279**
		327^22^	135^22^					104^22^							
2	*P*4_2_/*mnm*	**207**	**140**	**126**			**373**	**84**		**177**	**0.29**	**1.65**	**215**	**84**	**170**
		199^22^	131^22^	126^22^			389^22^	86^22^		180^22^	0.27^22^		211^22^	81^22^	167^22^
3	*I*4/*m*	**101**	**90**	**62**			**270**	**55**		**25**	**0.36**	**9.77**	**80**	**30**	**96**
4	*Imma*	**374**	**126**	**126**	**351**	**–3**	**351**	**84**	**34**	**84**	**0.29**	**1.72**	**216**	**84**	**170**
5	*Pnnm*	**209**	**142**	**127**	**210**	**128**	**376**	**84**	**85**	**178**	**0.29**	**1.72**	**216**	**84**	**172**
		215^22^	147	133	215	134	388	86	86	181					
6	*Pbcn*	**221**	**152**	**138**	**275**	**117**	**327**	**107**	**83**	**129**	**0.30**	**1.80**	**222**	**86**	**181**
		241^22^	154^22^	121^22^	256^22^	83^22^	257^22^	74^22^	92^22^	111^22^					
7	*Pbca*	**325**	**88.3**	**88.4**	**314.1**	**127.7**	**333.9**	**78.2**	**68.6**	**55.0**	**0.32**	**0.33**	**365**	**138**	**347**
		329^22^	164^22^	135^22^	353^22^	127^22^	346^22^	61^22^	82^22^	75^22^					
8	*Pnma-I*	**587**	**307**	**297**	**489**	**261**	**520**	**112**	**87**	**196**	**0.39**	**0.32**	**333**	**123**	**397**

a The modulus is in GPa.

The computed elastic constants are displayed in Table 2. All of the 11 polymorphs that satisfy structural stability and 8 polymorphs that met the dynamical stability; hence, we studied the mechanical stability criteria of the 8 SnO_2_ polymorphs from the elastic constants. *Imma* is a mechanically unstable phase, which is observed from the Born’s stability criteria. In total, seven polymorphs met all of the stability criteria and may form during the experimental synthesis. The bulk modulus calculated by BM-EOS (*B*_0_) is comparable with that of the values calculated from both the Voigt–Reuss–Hill approximations (*B*_H_) and experimental data.^22,32,35^ According to the Pugh criterion, the *B*/*G* ratio identifies the ductility/brittleness, which is an important mechanical factor for gauging the plastic deformation and braking ability of a material.^57^ While the Young’s modulus measures the stiffness of the material, the shear modulus is a measure of its resistance to plastic deformation. Further, we can also study the shear modulus over the bulk modulus for all the polymorphs of SnO_2_ are ductile with *B*/*G* > 1.75 and ν > 0.26, which suggests that they are all ductile at zero pressure. Here, *B*/*G* and ν, the Poisson’s ratio, are greater than the critical values. The bulk modulus of the three equally stable polymorphs are approximately equal.^36^ The calculated moduli of the polymorphs do not follow any particular order, which scattered 2–20% from the reported values.^22^ In addition, *Pnma-I* and *Pbca* having the highest elastic nature among the stable polymorphs mean that they have strong resistance toward the applied pressure. Moreover, from the Vickers hardness test, it is evident that it is a weakly compressible material. The *B*/*G* values and the compressibility of the polymorphs are given in Table S2 in the Supporting Information. This study shows how SnO_2_ polymorphs react to mechanical energy and are more advantageous under force.

#### 2.6.2 Elastic Anisotropy

Almost all crystalline solids are anisotropic, which means that physical quantities and orientations vary within the same crystal. This crystal anisotropy is relevant to a wide range of crystalline properties, including optical, magnetic, dielectric, and surface properties. Researchers are mostly interested in understanding crystal anisotropy and being able to control and predict crystal anisotropy, especially in the pharmaceutical industry. In the present work, the anisotropic properties of the SnO_2_ polymorphs can be determined from universal anisotropic index *A*^u^ and shear anisotropic indexes *A*_1_, *A*_2_, and *A*_3_, from the following equations.^58^
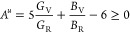
5where, *G*_V_ and *B*_V_ are shear and bulk moduli obtained from the Voigt approximation, respectively.
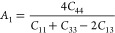
6
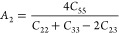
7
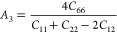
8

In general, the quantitative measurement of *A*^u^ specifies the elastic anisotropy of a single crystal. Normally, for an isotropic solid, *A*^u^ is equal to zero. Therefore, any deviation of *A*^u^ from zero signifies the degree of anisotropy. Hence, the value of *A*_1_, *A*_2_, and *A*_3_ is unity for an isotropic solid.

Table S3 of the Supporting Information contains the anisotropic indices of seven stable polymorphs of SnO_2_. The values of *A*_1_, *A*_2_, and *A*_3_ show the degree of anisotropy along the *xy*, *xz*, and *yz* planes, respectively. It is obvious that the *I*4/*m* polymorph is much more anisotropic as they have strongly deviated from the isotropic value. *P*4_2_/*mnm*, *Pnn*m, and *Pbcn* come next to *I*4/*m*, with finally *Pa*3̅ having the lowest anisotropic index.

**Figure 8:**
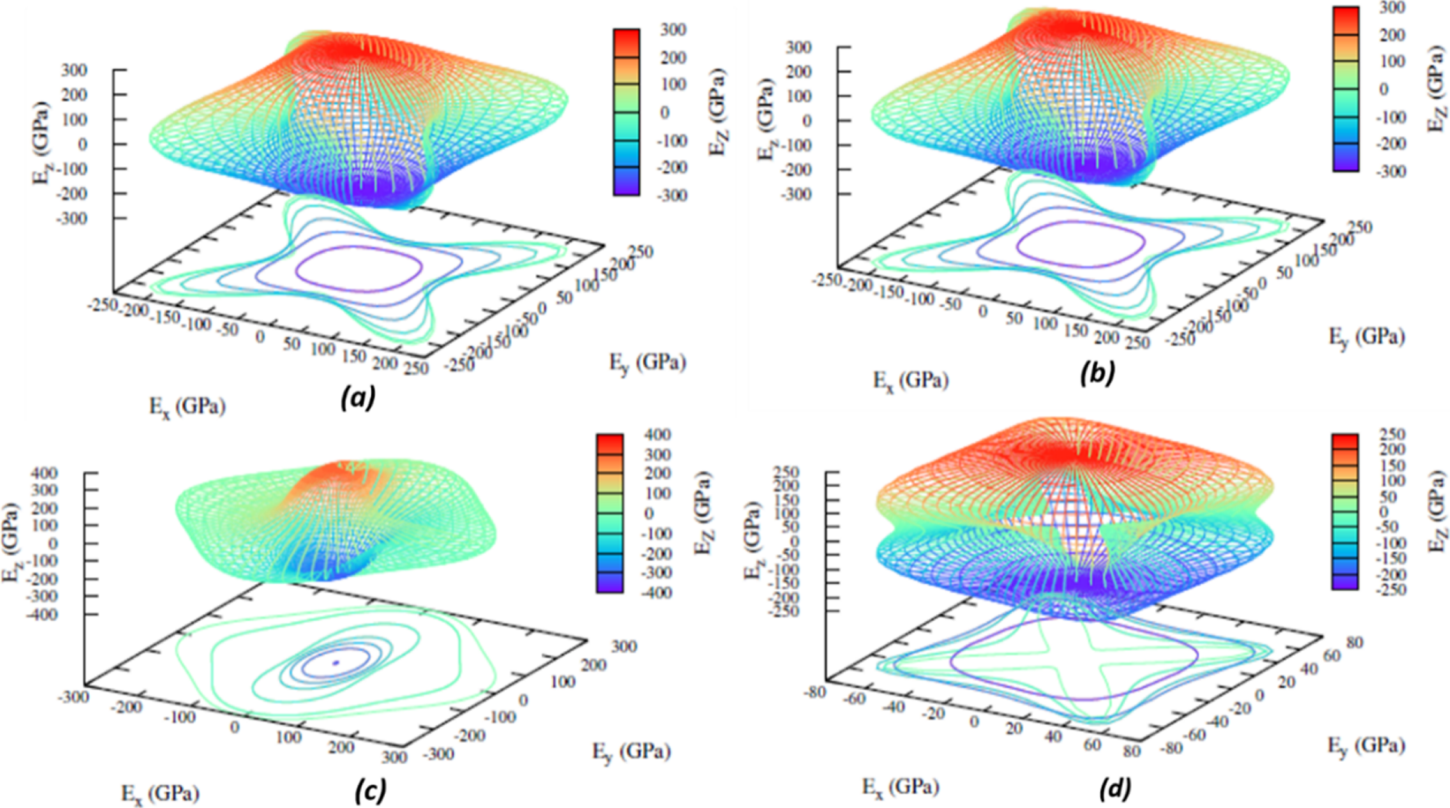
3D spatial dependence of Young’s modulus of the three minimum energy polymorphs (a) *P*4_2_/*mnm*, (b) *Pnnm*, (c) *Imma* and of (d) *I*4/*m*, the newly identified expended lattice polymorph of SnO_2_. The rest of the polymorphs are seen in the S3.

Usually, in an isotropic material, the 3D surface becomes spherical, and any deviation from the perfect sphere indicates higher anisotropy. Therefore, Figure 8 provides a comprehensive 3D visualization of the anisotropic properties of the SnO_2_ polymorphs for the orientation-dependent Young’s modulus along the *xy*, *xz*, and *yz* planes under ambient conditions for the minimum energy polymorphs with a newly identified *I*4/*m* polymorph. The anisotropic orientations of the minimum energy polymorphs *P*4_2_/*mnm* and *Pnnm* are similar. Because of their instability in structure and mechanical and thermodynamic properties, *Fm*3̅*m* and *I*4_1_/*amd* deviate from the sphere. In addition, from the compressibility values, *Fm*3̅*m* and *I*4_1_/*amd* are the most anisotropic polymorphs, which are omitted due to instability. Figure S3 of the Supporting Information shows that the spatial-dependent Young’s modulus of (a) *Pa*3̅ and (e) *Pbca* exhibits a spherical shape, presenting a homogeneous nature, while the rest of the polymorphs have multiple valleys indicating inhomogeneity. Additionally, the shear anisotropy and Poisson’s ratio of the polymorphs along the planes *xy*, *xz*, and *yz* are given in Figures S4 and S5 of the Supporting Information*.* Specifically, from the results of Vicker’s hardness test, it is inferred that *Pnma-I* is the strongest phase.

### 2.7 Raman and IR Vibrational Studies of SnO_2_ Polymorphs

**Figure 9:**
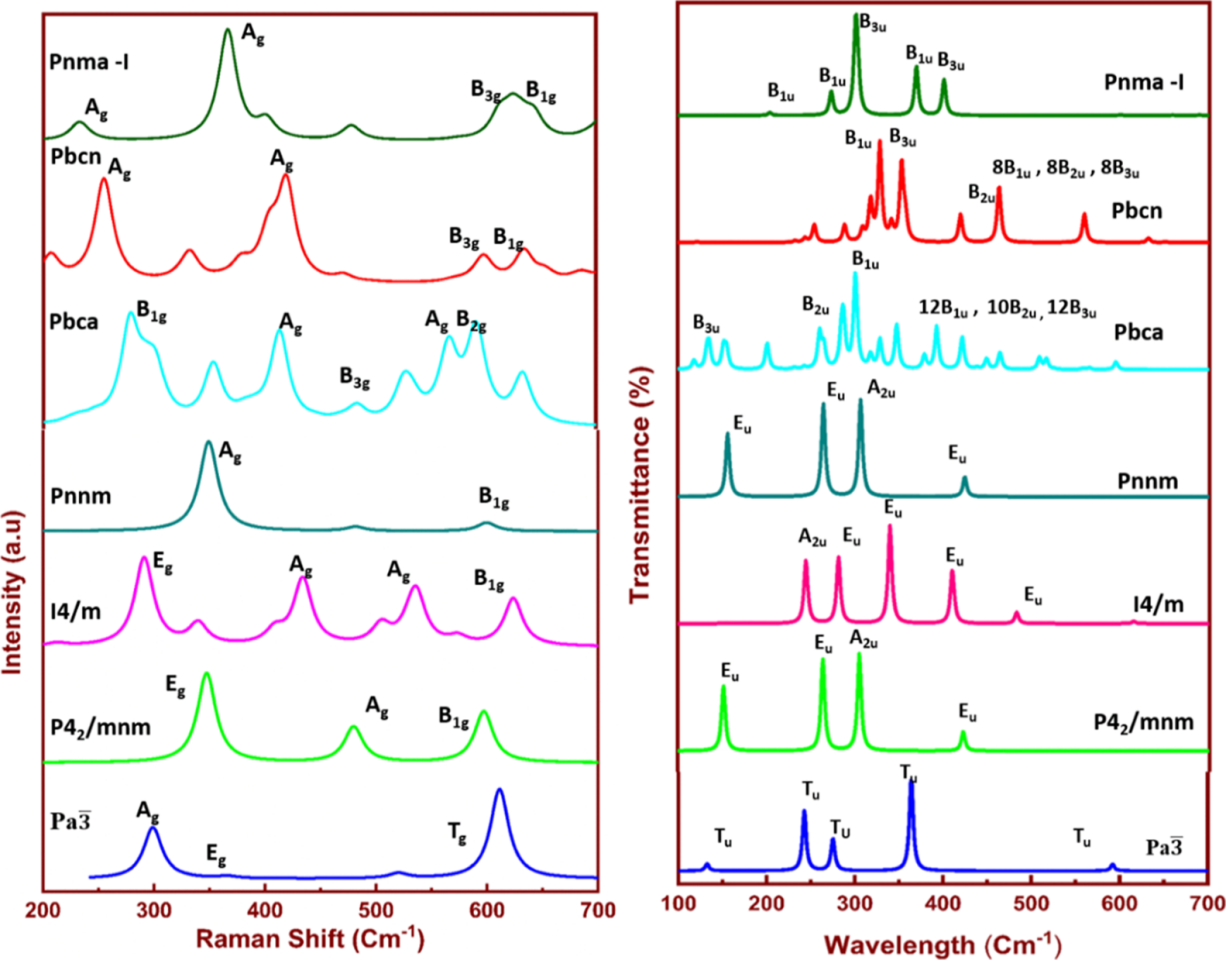
IR and Raman vibrational spectra of the stable polymorphs involved in the calculation with their vibrational assignments.

The *Imma* polymorph is mechanically unstable, and *Fm*3̅*m*, *Pnma*-II, and *I*4_1_/*amd* are dynamically unstable from phonon studies; hence, we investigated the vibrational studies of the remaining seven stable polymorphs. The Raman and IR activity of the seven crystalline polymorphs of SnO_2_ can be identified from the irreducible representation. The mode of vibration at zone centers in the Brillouin zone of the respective polymorphs are given in Figure S5 and Table S4. From these representations, it is observed that the orthorhombic structures exhibit more vibrations than other cubic and tetragonal structures. Although the five crystals have orthorhombic structures, due to different molecular structures and different lattice constants, their vibrational frequencies are different. We have calculated the Raman and IR frequencies of all the stable polymorphs of SnO_2_ as shown in Figure 9.

A complete description of the calculated Raman and IR frequencies are tabulated in Table S4 with the corresponding modes of vibrations. The suffix g in the irreducible representations represents the Raman active and u represents the IR active modes. The modes A_g_ and B_1g_ and B_2g_ and B_3g_ correspond to the vibrations in a plane perpendicular to the *c*-axis, whereas mode E_g_ corresponds to vibrations in the direction of the *c* axis. The two orthorhombic structures *Pbcn* and *Pbca* show similar modes of vibrations but their frequencies are different, which belong to the *D*_2*h*_ point group, and their structures are different. In addition, there is a shift in the Raman frequencies, which is observed for all the modes of these structures. The double degenerated E_g_ mode was observed in all the cubic, tetragonal, and *Pnnm* of the orthorhombic structures. Particularly, this E_g_ mode corresponds to vibrations of O atoms in the direction parallel to the *c* axis. In the Raman active modes, the O atom vibrates with respect to the Sn atom, whereas A_g_ and B_2g_ correspond to the expansion and contraction of the Sn–O bond. The B_1g_ mode has the rotation of the O atom around the Sn atom. The Raman peaks differ for different structures and offer an efficient way to differentiate the various forms of polymorphism. In the orthorhombic structures, the intensity of the A_1g_ mode of vibrations is prominent, whereas, in the tetragonal structures, the intensity of the E_g_ mode is prominent. When compared to the remaining structures, *Pbcn* has a greater number of absorption frequencies.

## 3 Conclusions

We used first-principles calculations to investigate 11 distinct SnO_2_ polymorphs so as to determine their relative stability, mechanical stability, and dynamical stability for the first time. Extensive research demonstrates the following: rutile is the global minimum structure among the identified polymorphs based on E–V data, also the new polymorph *I*4/*m* in the expanded lattice may form during the nanosynthesis of SnO_2_. The possible other metastable polymorphs *I*4_1_/*amd*, *Pbcn*, *Pnma-II*, and *I*4/*m*-SnO_2_ have been identified; however, under certain experimental conditions, these polymorphs may be stable. Furthermore, *Fm*3̅*m*, *I*4_1_/*amd*, and *Pnma-II* are unstable in the dynamical stability criteria, so we omitted these structures due to their imaginary frequency and may not be used to synthesize even under experimental conditions. The electronic structural studies of eight dynamically stable polymorphs were reported; from this, it is proved that all the structurally and dynamically stable SnO_2_ polymorphs were semiconductors obtained from the hybrid XC functional HSE06, which overcomes the underestimation of the band gap of the polymorphs by other theoretical reports. All polymorphs under this study are wide band gap semiconductors, which are inferred from the band gap values. *Pa*3̅, *Pbca*, and *Pnma-I-*SnO_2_ have an indirect band gap, while the rest of the stable polymorphs have a direct band gap. It is suitable for photocatalytic and photovoltaic gas sensors and also as a chemical sensor because its band gap scatters from 2.15 to 3.38 eV. The ionic bonding nature of the polymorphs makes them suitable for use as a base material in a variety of applications such as window materials in solar cells. *Imma* is a mechanically unstable phase; hence, it is not suitable for experimental synthesis. The orthorhombic *Pbca* (ZrO_2_) is the stiffest material because their shear modulus is the highest among the stable polymorphs. The high *B*/*G* values of all the polymorphs indicate that they are ductile in nature. Specific properties such as Young’s modulus, shear modulus, and Poisson’s ratio are studied. From this, it is concluded that *I*4_1_/*amd* has the most deviated in Young’s modulus, whose contour has several valleys. Our work has led us to conclude that 7 of the 11 polymorphs met all of the stability conditions, with rutile being the experimentally proven phase and *I*4/*m* the newly identified polymorph of SnO_2._ The newly found polymorphs may have evolved during the synthesis of the SnO_2_ nanostructure.

## 4 Methodology

Total energies were calculated by the projected APW implementation of the Vienna ab initio simulation package (VASP).^59−62^ These calculations were made with the Perdew, Burke, and Ernzerhof (PBE) exchange–correlation functional.^63^ The interaction between the core and the valence electrons was described using the PAW method.^64,65^ Ground-state geometries were determined by minimizing stresses and Hellman–Feynman forces using the conjugate gradient algorithm with a force convergence of less than 10^–3^ eV Å^–1^. Brillouin zone integration was performed with a Gaussian broadening of 0.1 eV during all relaxations. From various sets of calculations, it was found that 512 ***k***-points in the whole Brillouin zone for the structure with a 600 eV plane wave cutoff are sufficient to ensure optimum accuracy in the computed results. The *k*-points were generated using the Monkhorst–Pack method with a grid size of 8 × 8 × 8 for structural optimization. A similar density of ***k***-points and energy cutoff is used to estimate the total energy as a function of volume for all the structures considered in the present study as given in Table S5 of Supporting Information. Iterative relaxation of atomic positions was stopped when the change in the total energy between successive steps was less than 1 meV/cell. For improving the electronic energy level, the HSE (Heyd–Scuseria–Ernzerhof) exchange–correlation functional is used. This approach will provide accurate results that are comparable with experimental measurements.

A frozen phonon calculation was performed using suitable supercell models, using the Phonopy software to calculate the phonon dispersion and the associated density of states.^39^ The suitable supercell models are given in Table S5 of Supporting Information. A displacement of 0.0075 Å was applied to the atoms, with a symmetry consideration, to obtain the force constant matrix. Displacements along the opposite directions were included to improve the accuracy. The dynamical matrices were calculated from the force constants, and phonon density of state (PhDOS) curves were computed on a Monkhorst–Pack grid.^66^

The Raman and IR spectra for all the polymorphs of SnO_2_ are obtained from density functional perturbation theory as implemented in the CASTEP package.^67^ For the CASTEP computation, we have used the optimized VASP structures with a similar ***k*-**point mesh as the input with Norm-conserving pseudopotentials (energy cutoff of 800 eV) and the GGA exchange correlation functional proposed by PBE. Full geometry optimization was made, and we found that both codes gave almost similar lattice parameters and atomic positions.

## Supporting Information Available

Dielectric and mechanical property data in detail, Raman and IR frequencies, ***k***-grid and supercell size of SnO_2_ stable polymorphs, phonon dispersion relation and band structure of polymorphs, and spatial-dependent mechanical properties of the polymorphs (PDF)
