# Supplementary material for: First-Principles Exploration into the Physical and Chemical Properties of Certain Newly Identified SnO2 Polymorphs
Source: ACS Omega. 2022 Mar 16;7(12):10382–93. doi: 10.1021/acsomega.1c07063 (PMC8973149; doi:10.1021/acsomega.1c07063)
Supplement: Supplementary file 1 — ao1c07063_si_001.pdf [file ao1c07063_si_001.pdf]

Supplementary information of "A first-principles exploration into the physical and chemical properties of certain newly identified SnO_2 polymorphs"

Kanimozhi Balakrishnan[1], Vasu Veerapandy[1], Helmer Fjellvåg[2] and Ponniah Vajeeston[2]*

[1]*Department of Computational Physics, School of Physics, Madurai Kamaraj University, Palkalai Nagar, Madurai 625021, Tamil Nadu, India.*

[2] *Center for Materials Science and Nanotechnology, Department of Chemistry, University of Oslo, Oslo 0371, Norway*

* Electronic address: ponniahv@kjemi.uio.no; https://folk.universitetetioslo.no/ponniahv/

1. Dielectric constant

The static dielectric constant and BCE of the SnO_2 polymorphs for the low energy polymorphs were given blow.

Table S1. BEC of Sn and O atom along three axes and the static dielectric constant of the atom

**S.No**	**polymorphs**	**Static dielectric constant**	**BEC of O atom along a-axis**	**O atom along *b*-axis**	**O atom along *c*-axis**	**Sn atom along *a*-axis**	**Sn atom along *b*-axis**	**Sn atom along *c*-axis**
1	$Pa\bar{3}$	17.315	-2.039	-2.039	-2.039	4.078	4.078	4.078
2	$P4_2/mnm$	9.817<br>7.977	-1.920	-1.920	-2.044	3.841	3.841	4.088
3	*I4/m*	8.642<br>12.412	-2.497	-1.118	-2.201	3.682	3.267	4.263
4	*Imma*	9.817<br>7.977	-1.920	-1.920	-2.044	3.841	3.841	4.088
5	*Pnnm*	9.827<br>9.816<br>7.976	-1.920	-1.921	-<br>2.044	3.840	3.842	4.088
6	*Pbcn*	9.769<br>9.390<br>9.071	-1.899	-1.970	-2.021	3.848	3.944	4.047
7	*Pbca*	---	-2.081	-1.962	-2.157	4.088	4.019	4.094
8	*Pnma – I*	7.341<br>7.349	-1.301	-2.443	-1.938	3.748	3.747	3.878

		7.330						

2. Phonon dispersion

The phonon dispersion relation of the rest of the polymorphs were given *in Figure S1*.

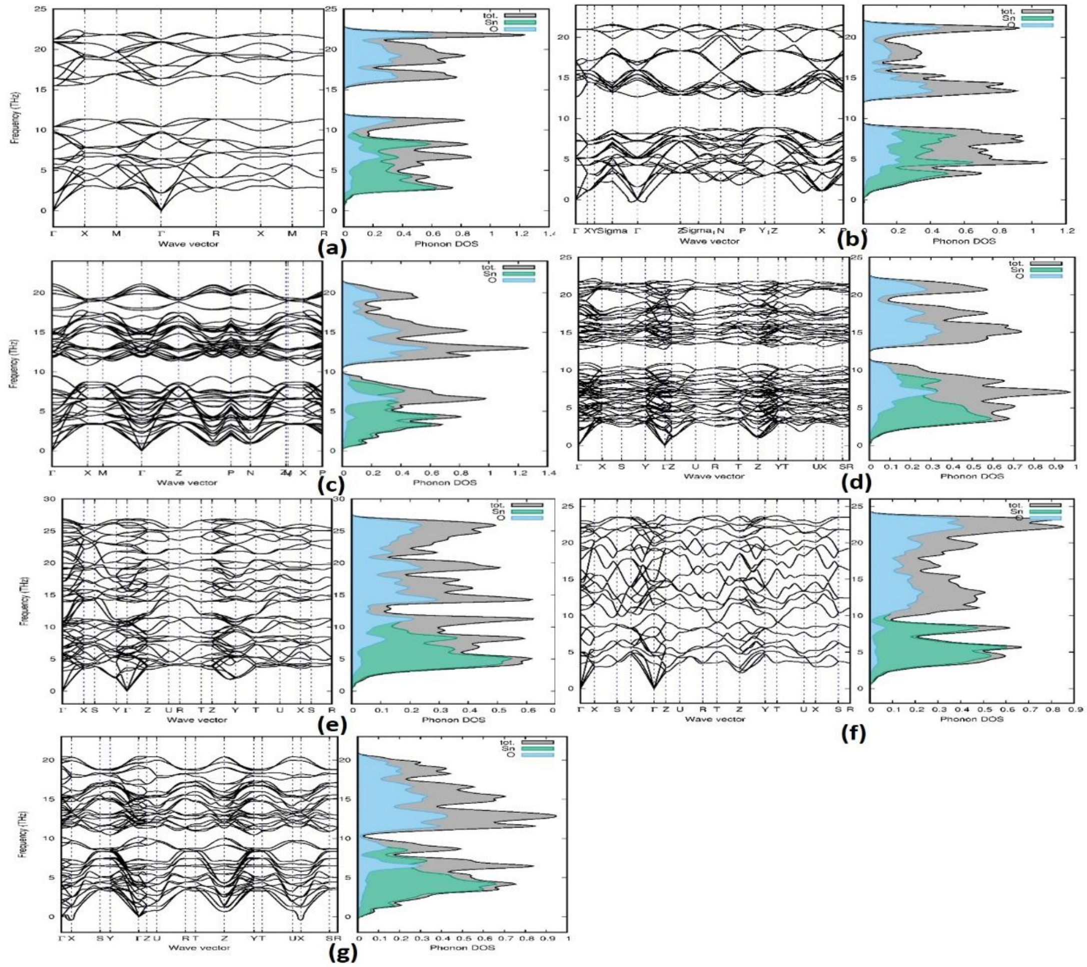

Figure S1. Phonon dispersion curve along with the Phonon density of states of 7 structurally low energy polymorphs (a) *Pa3,* (b) *I4$_1$/amd,* (c) *I4/m,* (d) *Pbcn,* (e) *Pnca,* (f) *Pnnma-I,* (g) *Pnma-II.*

3. Band structure

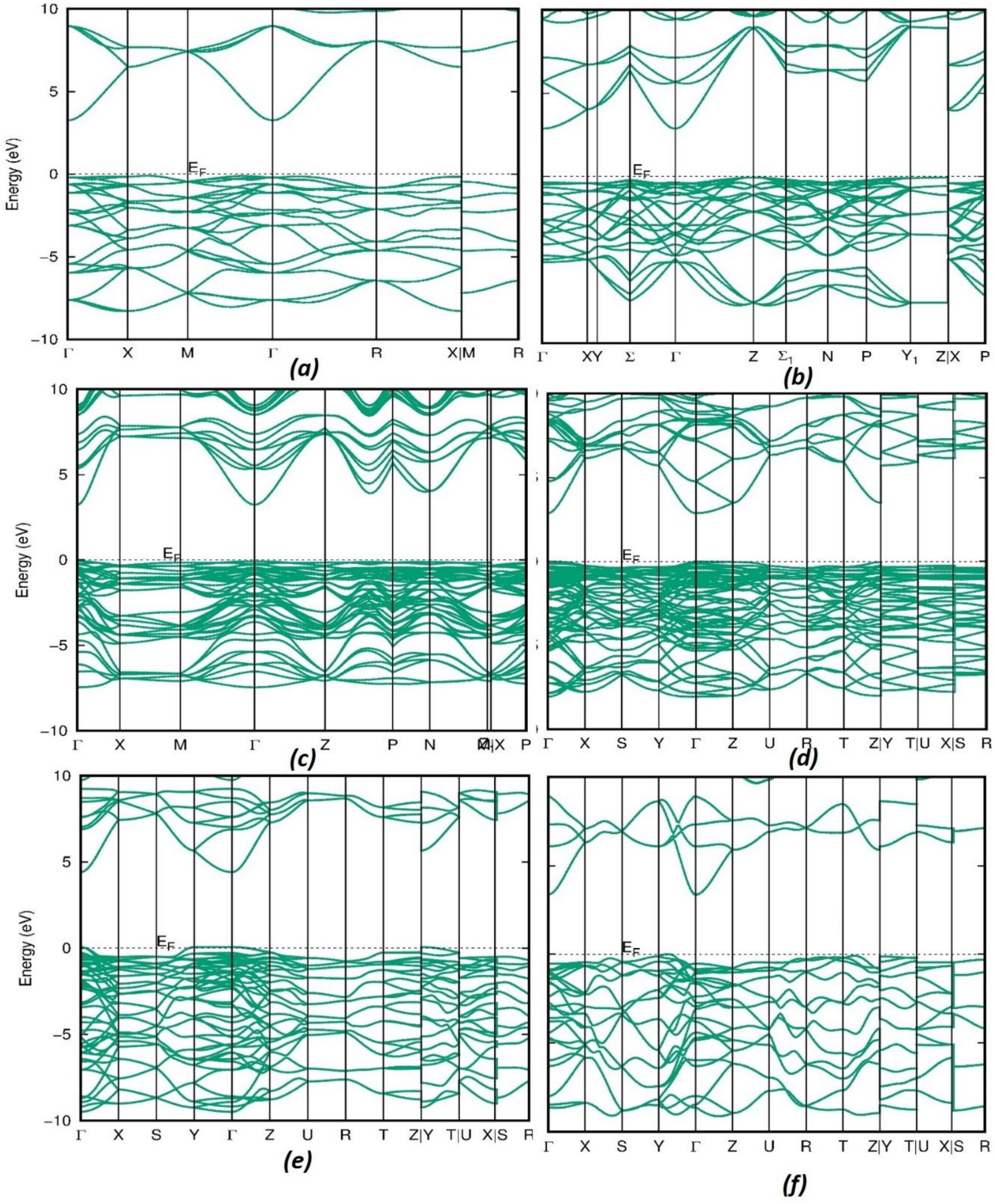

Figure S2. Band structure of low energy polymorphs of SnO_2 (a) *Pa*$\bar{\mathbf{3}}$, (b) *I4$_1$/amd,* (c) *I4/m,* (d) *Pbcn,* (e) *Pnca,* (f) *Pnnma-II.*

4. **Mechanical properties**

Table S2. The calculated Young's modulus (E), shear modulus (G), Bulk modulus (B_H) Poisson's ratio, Vicker's Hardness and compressibility of the SnO_2 Polymorphs.

**S.No**	**Space group**	**E (GPa)**	**G (GPa)**	**B_H**	**B_H/G**	**σ**	**Vickers hardness**	**Compressibility TPa^{-1}**
1	$Pa\overline{3}$	317	121	279	2.31	0.31	85	3.59
2	*P4₂/mnm*	215<br>211[22]	84<br>81[22]	170<br>167[22]	2.02	0.29<br>0.27[22]	58	5.87
3	*I4/m*	80	30	96	3.2	0.36	54	10.46
4	*Pnnm*	216	84	172	2.05	0.29	59	5.80
5	*Pbcn*	222	86	181	2.12	0.30	62	5.53
6	*Pbca*	365	138	347	2.52	0.32	102	2.88
7	*Pnma - I*	126	49	101	2.06	0.29	43	9.85

Table S3. Universal Anisotropic index A^u and shear anisotropic index A_1, A_2, A_3 of SnO_2 Polymorphs.

**S.No**	**Space group**	**G_v**	**G_R**	**Bv**	**Br**	**A^u**	**A_1**	**A_2**	**A_3**
1	$Pa\overline{3}$	122	120	279	279	0.083	1.288	1.288	1.288
2	$P4_2/mnm$	95	72	175	166	1.651	1.024	1.024	5.284
3	*I4/m*	44	15	100	91	9.765	0.891	0.891	4.545
4	*Pnnm*	96	72	177	168	1.721	1.015	1.030	5.274
5	*Pbcn*	92	80	182	180	1.804	1.574	0.902	2.688
6	*Pbca*	142	134	352	342	0.328	0.940	1.756	1.286
7	*Pnma-I*	128	119	370	365	0.392	0.873	0.715	1.697

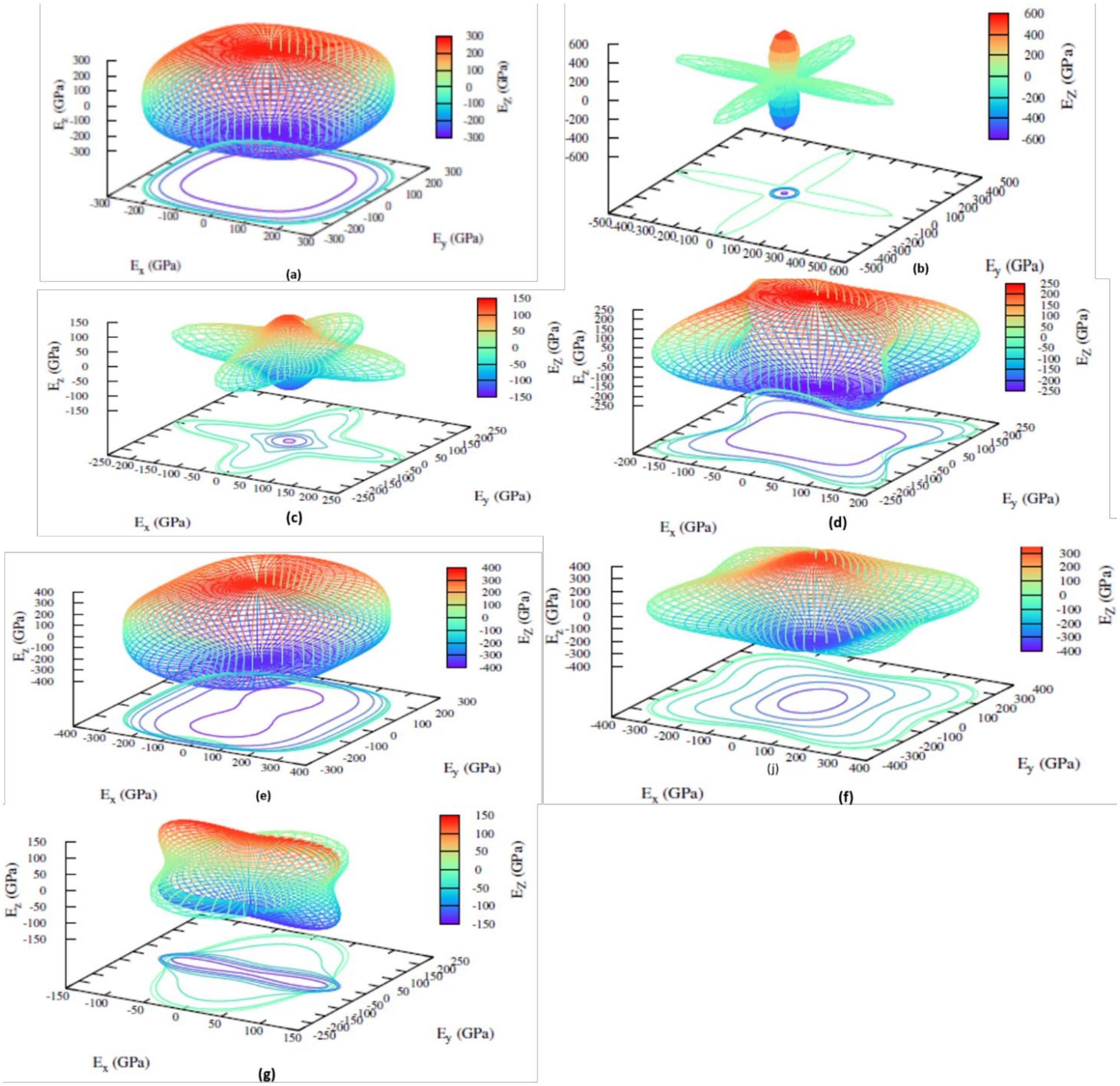

Figure S3. The 3D spatial dependence of the Young modulus of SnO_2 polymorphs (a) *Pa*$\overline{3}$, (b) *Fm* $\overline{3}$*m*, (c) *I*4_1*/amd* (d) *Pbcn* (e) *Pbca* (f) *Pnma-I* (g)*Pmna –II.*

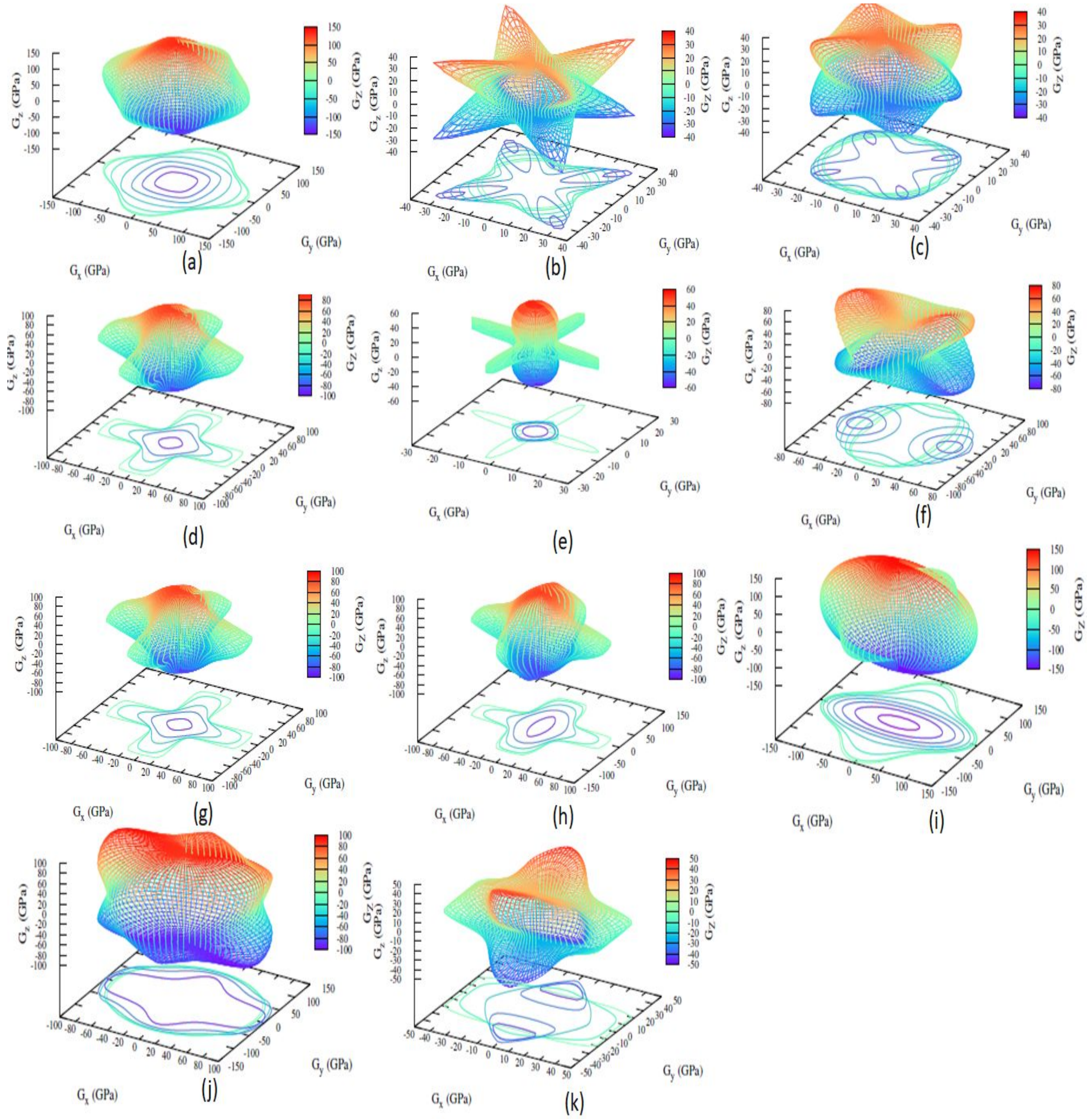

Figure S4. The 3D spatial dependence of the shear modulus (G) of SnO_2 polymorphs(a) $Pa\bar{3}$, (b) $Fm\bar{3}m$, (c) $I4_1/amd$, (d) $P4_2/mnm$ (e) *I4/m* (f) *Imma* (g) *Pnnm* (h) *Pbcn* (i) *Pbca* (j) *Pnma* –I (k) *Pmna- II.*

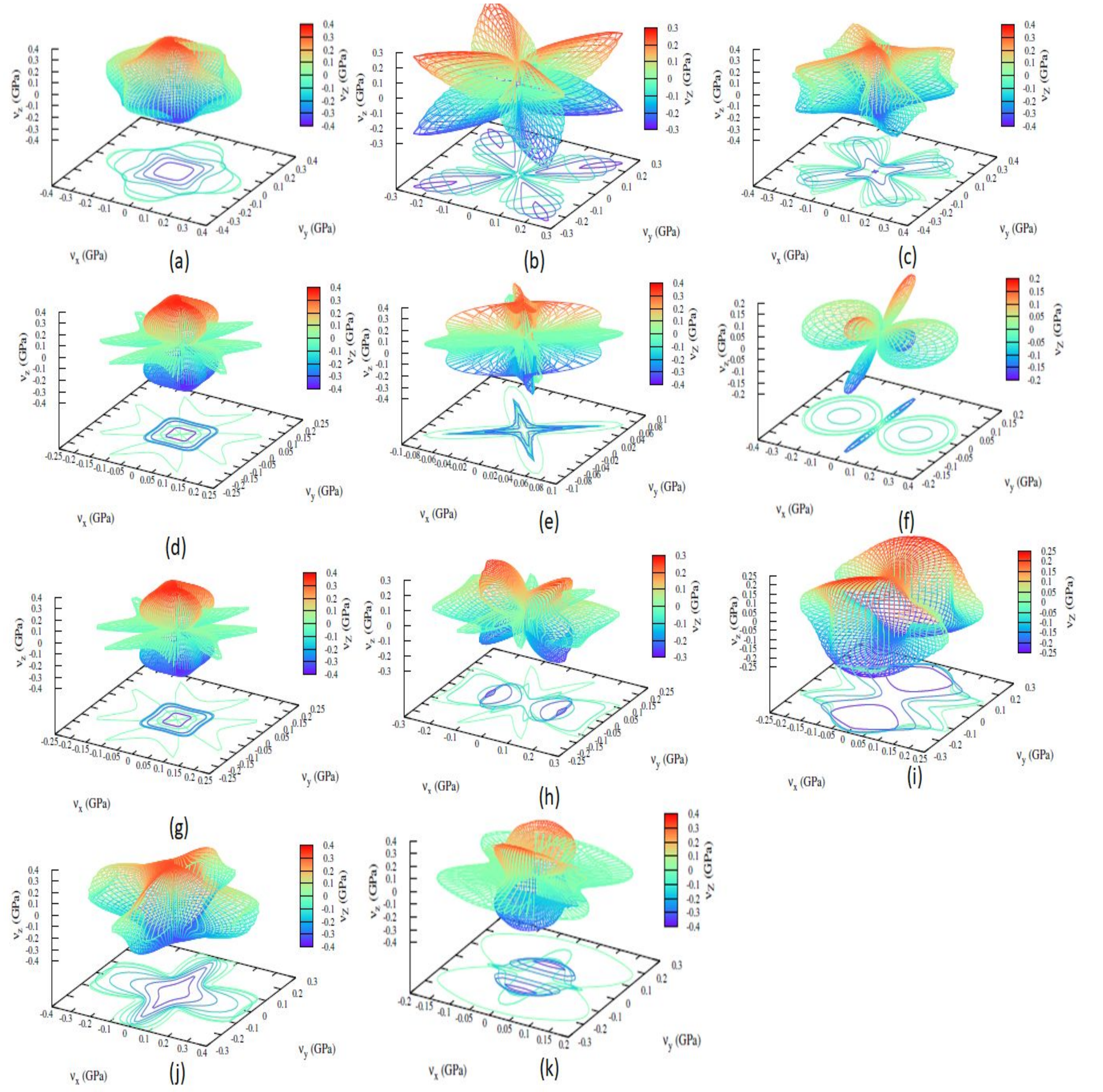

Figure S5. The 3D spatial dependence of the Poisson's ratio of SnO_2 polymorphs(a) *Pa*$\overline{3}$, (b) *Fm* $\overline{3}$*m*, (c) *I4*$_1$*/amd*, (d) *P4*$_2$*/mnm* (e) *I4/m* (f) *Imma* (g) *Pnnm* (h) *Pbcn* (i) *Pbca* (j) *Pnma-I* (k) *Pmna –II*.

5. Raman and IR vibrational studies of SnO_2

The irreducible representation of the eleven phases of SnO_2 along with group theory has given below. R= Raman and I = IR active.

(Pa$\overline{3}$) : $\Gamma_{optic} = A_g (R) + {}^1E_g (R) + {}^2E_g (R) + 3T_g (R) + 5Tu (I)$

$P4_2/mnm$ (136) : $\Gamma_{optic} = A_g (R) + B_{1g} (R) + B_{2g} (R) + E_g (R) + A_{2u} (I) + 3E_u (I)$

I4/m (87) : $\Gamma_{optic} = 6A_g (R) + 6B_g (R) + 3{}^1E_g (R) + 3{}^2E_g (R) + 2A_u (I) + 5{}^1E_u (I) + 5{}^2E_u (I)$

Pnnm (58) : $\Gamma_{optic} = A_g (R) + B_{1g} (R) + B_{2g} (R) + E_g (R) + A_{2u} (I) + 3E_u (I)$

Pbcn (60) : $\Gamma_{optic} = 11A_g (R) + 13B_{1g} (R) + 11B_{2g} (R) + 13B_{3g} (R) + 12B_{1u} (I) + 10B_{2u} (I) + 12B_{3u} (I)$

Pbca(61) : $\Gamma_{optic} = 9A_g (R) + 9B_{1g} (R) + 9B_{2g}(R) + 9B_{3g} (R) + 8B_{1u} (I) + 8B_{2u} (I) + 8B_{3u} (I)$

Pnma-I (62) : $\Gamma_{optic} = 12A_g (R) + 6B_{1g} (R) + 12B_{2g} (R) + 6B_{3g} (R) + 11B_{1u} (I) + 5B_{2u} (I) + 11B_{3u} (I)$

Table S4. The computed Raman and IR modes of SnO_2 polymorphs at zone centers.

Γ	(Pa$\overline{3}$)	Γ	$P4_2/mnm$	Γ	I4/m	Γ	Pnnm	Γ	Pbcn	Pbca	Pnma-I
A_g	299	A_g	479	A_g	112 212 339 433 504 536	A_g	481	A_g	49, 79 129, 223 232, 278 301, 381 413, 483 525, 566 591	128,148 206, 254 332, 403 419, 598 736	139 231 366 479 624 720
E_g	366	B_{1g}	596	B_g	147 157 295 408 529 623	B_{1g}	599	B_{1g}	37, 78 133, 169 247, 292 314, 354 375, 391 471, 527 582, 633	165, 192 232, 236 387, 497 593, 634 809	158 476 691
T_g	327 519 611	B_{2g}	222	E_g	140 291 573	B_{2g}	223	B_{2g}	47, 122 167, 268 305, 347 352, 403 444, 465 561, 593 633	107, 177 309, 322 378, 577 653, 687 770	236 300 401 612 644 705
T_u	132 242 275 364 592	E_g	347	E_u	160 281 339 482 614	E_g	349	B_{3g}	55, 107 133, 167 223, 247 276, 290 390, 448 484, 533 566, 605	183, 197 273, 359 471, 570 680, 725 757	141 574 632

		A2u	304	A_{2u}	244	A2u	306	B_{1u}	43, 118<br>151, 231<br>242,,264<br>300, 347<br>392, 437<br>508, 523<br>564	0 ,172<br>232, 328<br>341, 461<br>559, 710<br>763	204<br>303<br>400<br>599<br>725
		Eu	152 264<br>422			Eu	156<br>265<br>424	B_{2u}	78, 133<br>155, 260<br>287, 328<br>370, 421<br>463, 524<br>553, 594	0, 121<br>308, 317<br>357, 462<br>631, 776	299<br>580
								B_{3u}	75, 135<br>151, 200<br>284, 301<br>317, 347<br>378, 448<br>516, 557<br>594	243, 254<br>288, 352<br>419, 650<br>707	75 273<br>369<br>658<br>688

6. Structure models

Table S5. Used **k**-grid size for the HSE calculation and supercell size for the phonon calculation for the SnO_2 stable polymorphs.

**Polymorphs**	**k-grid**	**Supercell size**
$Pa\overline{3}$ (205)	8 x 8 x 8	2 x 2 x 2
$Fm\overline{3}m$ (225)	8 x 8 x 8	2 x 2 x 2
$I4_1/amd$ (141)	4 x 4 x 10	3 x 3 x 1
$P4_2/mnm$ (136)	8 x 8 x 10	2 x 2 x 3
I4/m (87)	4 x 4 x 12	1 x 1 x 3
Imma (74)	8 x 8 x 8	2 x 2 x 2
Pnnm (58)	8 x 8 x 10	2 x 2 x 1

Pbcn (60)	8 x 6 x 3	2 x 2 x 1
Pbca (61)	8 x 4 x 8	2 x 1 x 2
Pnma –I (62)	8 x 10 x 4	2 x 3 x 2
Pnma – II (62)	4 x 12 x 4	1 x 3 x 1
